# Effect of continuing the use of renin–angiotensin system inhibitors on mortality in patients hospitalized for coronavirus disease 2019: a systematic review, meta-analysis, and meta-regression analysis

**DOI:** 10.1186/s12879-023-07994-7

**Published:** 2023-01-24

**Authors:** Qi Liu, Wei Fu, Chang-ju Zhu, Zi-heng Ding, Bin-bin Dong, Bao-qing Sun, Rong-chang Chen

**Affiliations:** 1 Translational Medicine Center, Emergency Intensive Care Ward, The First Affiliated Hospital of Zhengzhou University, 1st Jianshe East Road, Zhengzhou, 450052 Henan People’s Republic of China; 2 State Key Laboratory of Respiratory Disease, National Clinical Research Center for Respiratory Disease, Guangzhou Institute of Respiratory Health, The First Affiliated Hospital of Guangzhou Medical University, 151st Yanjiang Road, Guangzhou, 510120 Guangdong People’s Republic of China; 3 Department of Emergency Medicine, The First Affiliated Hospital of Zhengzhou University, Zhengzhou, 450052 Henan People’s Republic of China; 4 Department of Shenzhen Institute of Respiratory Diseases, Shenzhen People’s Hospital, Shenzhen, 518020 Guangdong People’s Republic of China

**Keywords:** Renin–angiotensin system inhibitors, COVID-19, Hypertension, Hospital mortality, Regression analysis

## Abstract

**Background:**

The effect of angiotensin-converting enzyme inhibitors (ACEIs)/angiotensin receptor blockers (ARBs) on mortality was preliminarily explored through the comparison of ACEIs/ARBs with non-ACEIs/ARBs in patients with coronavirus disease 2019 (COVID-19). Reaching a conclusion on whether previous ACEI/ARB treatment should be continued in view of the different ACE2 levels in the comparison groups was not unimpeachable. Therefore, this study aimed to further elucidate the effect of ACEI/ARB continuation on hospital mortality, intensive care unit (ICU) admission, and invasive mechanical ventilation (IMV) in the same patient population.

**Methods:**

We searched PubMed, the Cochrane Library, Ovid, and Embase for relevant articles published between December 1, 2019 and April 30, 2022. Continuation of ACEI/ARB use after hospitalization due to COVID-19 was considered as an exposure and discontinuation of ACEI/ARB considered as a control. The primary outcome was hospital mortality, and the secondary outcomes included 30-day mortality, rate of ICU admission, IMV, and other clinical outcomes.

**Results:**

Seven observational studies and four randomized controlled trials involving 2823 patients were included. The pooled hospital mortality in the continuation group (13.04%, 158/1212) was significantly lower than that (22.15%, 278/1255) in the discontinuation group (risk ratio [RR] = 0.45; 95% confidence interval [CI], 0.28–0.72; P = 0.001). Continuation of ACEI/ARB use was associated with lower rates of ICU admission (10.5% versus 16.2%, RR = 0.63; 95% CI 0.5–0.79; P < 0.0001) and IMV (8.2% versus 12.5%, RR = 0.62; 95% CI 0.46–0.83, P = 0.001). Nevertheless, the effect was mainly demonstrated in the observational study subgroup (P < 0.05). Continuing ACEI/ARB had no significant effect on 30-day mortality (P = 0.34), acute myocardial infarction (P = 0.08), heart failure (P = 0.82), and acute kidney injury after hospitalization (P = 0.98).

**Conclusion:**

Previous ACEI/ARB treatment could be continued since it was associated with lower hospital deaths, ICU admission, and IMV in patients with COVID-19, although the benefits of continuing use were mainly shown in observational studies. More evidence from multicenter RCTs are still needed to increase the robustness of the data.

*Trial registration* PROSPERO (CRD42022341169). Registered 27 June 2022

**Supplementary Information:**

The online version contains supplementary material available at 10.1186/s12879-023-07994-7.

## Background

The coronavirus disease 2019 (COVID-19) pandemic has imposed unprecedented challenges on both the worldwide health system and global economic development [1]. COVID-19 is a viral multiorgan disease although it predominantly originates from the respiratory system [2]. In particular, the renin–angiotensin–aldosterone system (RAAS) has been implicated, as it has not only been associated with the transmission of severe acute respiratory syndrome coronavirus 2 (SARS-CoV-2) through angiotensin-converting enzyme 2 (ACE2) [3], but also with inflammatory lung injury. During the development of COVID-19, RAAS plays multiple roles. On one hand, angiotensin-converting enzyme (ACE) converts angiotensin I (Ang I) into angiotensin II (Ang II), increases oxidative stress, promotes inflammation, and induces fibrosis through the type 1 angiotensin receptor (AT1R) [4, 5]. On the other hand, ACE2, another enzyme in the RAAS, transforms Ang II into Ang-(1–7), increases the level of nitric oxide, and alleviates inflammation by combining with the Mas receptor [6, 7]. In theory, ACE inhibitors (ACEIs)/angiotensin receptor blockers (ARBs) potentially relieve SARS-CoV-2-induced lung injury by downregulating the proinflammatory effect of the ACE-Ang II-AT1R pathway, thus redirecting considerable attention to the potential benefit of ACEIs/ARBs [8, 9]. Nonetheless, animal model studies have also indicated that ACEIs/ARBs could upregulate the expression and enhance the activity of ACE2 [10, 11], which plays an important role in the mechanism by which SARS-CoV-2 enters the human lungs [12, 13], and thereby potentially aggravates lung injury. As a result, there are two opposing hypotheses regarding the effect of ACEIs/ARBs on SARS-CoV-2-induced lung injury, that is, whether ACEIs/ARBs are protective or harmful [7, 14].

The results of certain clinical trials comparing patients taking ACEIs/ARBs with those receiving other antihypertensive agents revealed that ACEIs/ARBs were associated with a lower risk of mortality in patients with COVID‐19 and hypertension [15]; however, other studies found that ACEIs/ARBs did not affect mortality or severe diseases in a conservative tone [16–19]. In these studies, the levels of ACE2 in COVID-19 patients with and without the chronic use of ACEIs/ARBs were not completely identical; the expression of ACE2 in the former might have been regulated by the chronic use ACEIs/ARBs nevertheless, ACE2 in the latter was in the protoform state. In other words, there are two differences existed between the comparative groups: the intervention strategy (taking ACEIs/ARBs or not) and baseline ACE2 levels. Hence, an immediate conclusion regarding the continuation of ACEIs/ARBs in patients who have previously undergone chronic ACEI/ARB treatment may be premature, according to the aforementioned studies. Therefore, comparison of mortality between patients who continue and those who discontinue previous ACEI/ARB treatment is warranted [7]. Such a comparison potentially contributes to the elucidation of the feasibility of continued ACEI/ARB after a COVID-19 diagnosis [20–22]. This study aimed to further determine the effect of ACEI/ARB continuation on hospital mortality, ICU admission, and IMV compared with discontinuation of ACEI/ARB in the patients with the chronic use of ACEIs/ARBs before being infected with SARS-CoV-2.

## Methods

### General information

This systematic review, meta-analysis, and meta-regression analysis was performed under the guidance of the PRISMA statement [23]. The protocol was registered in the PROSPERO database (CRD42022341169). Ethical review and informed consent were waived for this type of study. In this study, participants comprised adult patients with COVID-19 who had a history of chronic ACEI/ARB therapy for the treatment of their underlying diseases. We considered the continuation of ACEIs/ARBs after hospitalization for COVID-19 as an exposure and the discontinuation of ACEIs/ARBs as a control. The primary outcome of interest was hospital mortality, and the secondary outcomes were 30-day mortality, rate of intensive care unit (ICU) admission, rate of invasive mechanical ventilation (IMV) use, acute myocardial infarction, new or worsening congestive heart failure, and new onset acute kidney injury after hospitalization.

### Search strategy

The electronic search was performed in PubMed, the Cochrane Library, and Ovid Embase for relevant articles published between December 1, 2019 and April 30, 2022. The following combinations of terms or keywords were used: (COVID-19 OR corona virus OR SARS-CoV-2) AND (ACE inhibitors OR ARB OR angiotensin-converting enzyme inhibitors OR angiotensin receptor blockers OR RAS inhibitors OR renin–angiotensin system inhibitors). We did not stipulate any further restrictions except the publication time. Duplicate papers were identified and counted once. The references of the relevant publications were verified manually to identify potentially eligible studies.

### Eligibility criteria

Full texts were evaluated for potentially eligible studies based on the inclusion and exclusion criteria by Q.L. and W.F. independently. In case of disagreement, they discussed and consulted a third author (C.Z.) for arbitration. The inclusion criteria were as follows: (1) participants aged ≥ 18 years, (2) COVID-19 hospitalization and diagnosis, (3) ACEI/ARB administration as a chronic treatment for any indication prior to COVID-19 infection, (4) at least one objective to compare the effect of continuing previously administered ACEIs/ARBs with that of discontinuing ACEIs/ARBs, and (5) at least one outcome of interest extractable from the publication and Additional file 1. The exclusion criteria included the following: (1) patients aged < 18 years, (2) trials comparing effect of ACEIs/ARBs with that of placebos or other antihypertensive agents, (3) sole availability of an abstract or meeting paper without published full text, and (4) non-original studies, such as editorials, case reports, reviews, and guidelines. Additionally, only one of the papers was included if multiple articles were published for the same trial.

### Data extraction and quality assessment of the included studies

The characteristics and outcomes of interest of the included studies were extracted and managed using a spreadsheet. The numbers of events, and the sample sizes in each group were directly extracted to acquire dichotomous data from the articles and appendixes. The Newcastle–Ottawa Scale (NOS) was used to assess the quality of cohort studies in this meta-analysis [24]. The NOS was used to evaluate quality with regard to three aspects: selection, comparability, and outcome. One star was awarded for each numbered item within the selection and outcome categories if it qualified, while a maximum of two stars was awarded for comparability. A maximum of nine stars could be awarded to a study. For randomized controlled trials (RCTs), the Cochrane Collaboration tool was adopted to assess the risk of bias [25]. This tool appraised the quality in six dimensions: sequence generation, allocation concealment, blindness to participants and personnel, outcome assessment, data integrality of the outcome, and selective reporting. Each item was classified as follows: low risk, high risk, or unclear risk of bias based on the related information. Two authors (Q.L. and W.F.) performed the evaluations independently. Disagreements were resolved by discussion and subsequently referred to a third author (C.Z.) for arbitration.

### Statistical analysis

We adopted risk ratios (RRs) with 95% confidence intervals (CIs) to determine the effect size of dichotomous outcomes in this study. The pooled RR was calculated using the Mantel–Haenszel method with the fixed model if the heterogeneity was not high (< 75%); otherwise, the random model was employed. Additionally, to get the adjusted RR and evaluate the stability of the result, the RRs were logarithmically transformed and then pooled together combined with the corresponding stand errors, which were calculated from 95% CIs of crude RR. Heterogeneity was estimated quantitatively using I square (I^2^) [26, 27], and the value of I^2^ was divided into three levels: < 50% indicated low heterogeneity, 50%-75% moderate heterogeneity, and ≥ 75% high heterogeneity [28]. Subgroup analyses were performed based on the research design (RCT or observational study) and number of research centers (single or multiple-centers). Sensitivity analyses were performed by removing each study one by one or changing the effect model to observe the changes in heterogeneity and check whether the results remained stable, especially after removing some low-quality studies. Univariate and multivariate meta-regression analyses based on random-effects restricted maximum likelihood were performed to estimate the effect of the characteristics of trials on the relationship between ACEIs/ARBs and hospital mortality. P < 0.05 was considered as a significant difference. We utilized both Stata/IC (version 16.1, Single-user License, StataCorp, Texas, USA) and Review Manager 5.3.5 (Cochrane Collaboration, Oxford, UK) for statistical analyses.

## Results

**Fig. 1:**
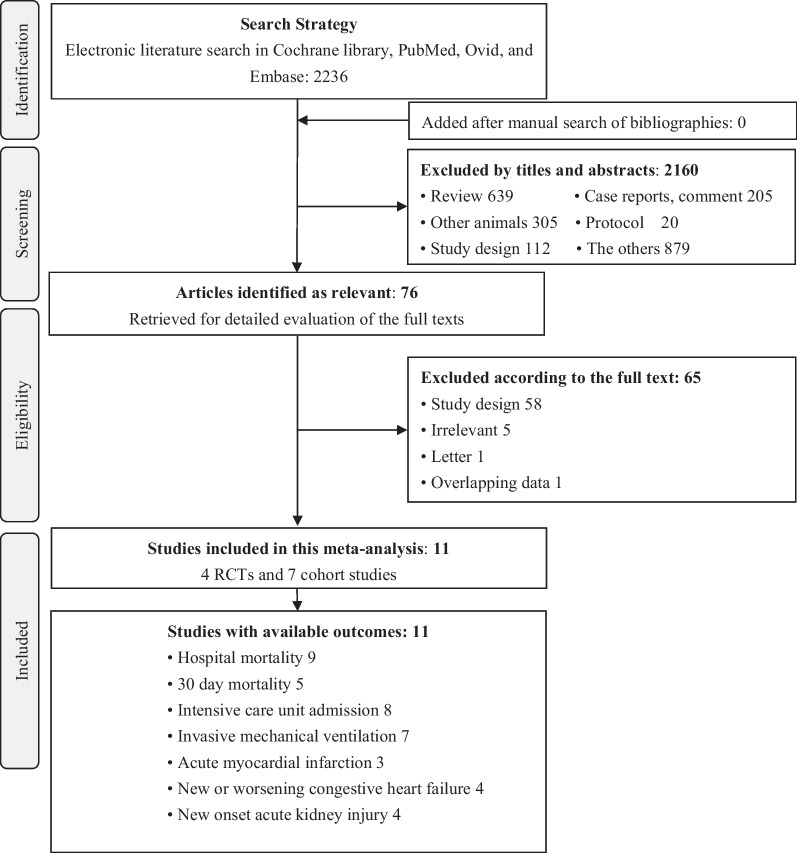
Flow chart of the trial inclusion in this study. *RCT* randomized controlled trial, *COVID-19* Corona Virus Disease 2019

As shown in Fig. 1, 2236 articles were identified from the electronic search. Thereafter, 2160 were excluded based on their titles and abstracts. Further full text evaluation eliminated 65 studies, including one study that reported overlapping data from a different perspective [29]. Finally, we included 11 studies, including seven observational studies [30–36] and four RCTs [37–40]. In total, 2823 hospitalized patients were recruited in the eleven studies. Five studies were performed in single centers and six in multiple centers. The studies were distributed across several countries, including Canada [37], United States [31, 32], Spain [35, 36], Italy [30], Brazil [40], Iran [33], France [34], Austria, and Germany [38]. One study simultaneously covered several American countries and Sweden [39]. More characteristics of the included studies are presented in Table 1. The reasons of discontinuing ACEI/ARB were summarized in Additional file 1: Table S1.

**Table 1:** Characteristics of the included studies in this study

Study year	Study design	Country	Sample		Demographics		Comorbidity (%)					ICU (%)
			Con	Dis	Age (Year)	Male (%)	CHD	HF	CKD	Diabetes	COPD	
Cannata et al. 2020 [30]	Prospective single-center observation	Italy	56	117	NA	NA	NA	NA	NA	NA	NA	NA
Lam et al. 2020 [31]	Retrospective single-center cohort study	America	164	171	68	56.4	24.6	11.3	9.0	45.4	NA	19.4
Chaudhri et al. 2020 [32]	Retrospective single-center cohort study	America	49	31	68.5	60.0	31.0	29.0	19.0	46.0	11.0	28.0
Soleimani 2020 [33]	Retrospective single center observational study	Iran	79	43	68	59.0	NA	NA	12.3	46.7	NA	NA
Lahens et al. 2021 [34]	Retrospective single center cohort study	France	78	39	65	59.0	17.0	13.0	26.0	49.0	6.0	20.5
Aparisi et al. 2022 [35]	Retrospective multi-center Non experimental comparative study	Spain	151	159	74	47.4	12.7	NA	11.0	26.8	9.6	12.0
de Abajo et al. 2021 [36]	Retrospective multi-center cohort study	Spain	285	340	74	60.6	15.8	11.0	11.5	74	60.6	5.8
Sharma et al. 2022 [37]	Prospective multi-center RCT	Canada	25	21	71.5	56.5	32.6	10.9	19.6	43.5	4.4	6.5
Bauer et al. 2021 [38]	Prospective multi-center RCT	Austria and Germany	100	104	75	63.2	22.0	9.0	18.1	32.8	15.7	23.0
Cohen et al. 2021 [39]	Prospective multi-center RCT	America, Canada, Mexico, Sweden, Peru, Bolivia, and Argentina	75	77	62	55.0	11.8	3.9	19.1	0.5	NA	19.7
Lopes et al. 2021 [40]	Pragmatic, registry based, randomized	Brazil	325	334	55.1	59.6	4.6	1.4	1.4	31.9	NA	NA

*Con* continuation, Dis* discontinuation, *CHD* chronic heart disease, *HF* heart failure, *CKD* chronic kidney disease, *COPD* chronic obstructive pulmonary disease, *ICU* intensive care unit, *RCT* randomized control trail

### Quality assessment of the included studies

The quality of the included trials was high in both RCTs and cohort studies. The bias in RCTs was rated as low risk for most of the appraisal items, while high risk of bias or unclear risk of bias was predominantly associated with the course of performance, that is, participants and personnel were not blinded for the safety of the patients (Table 2). All the included cohort studies were awarded at least five stars, and half of them were awarded eight stars, thus yielding a high quality of cohort studies (Table 3). In the cohort studies, all the patients were confirmed using clear diagnostic criteria, and they originated from the same population. Simultaneously, the continuation or discontinuation of ACIEs/ARBs was described explicitly or judged according to the electronic medical record system, and stars were awarded for the selected items and for comparability among all the included studies. Outcome assessment was awarded a star if the mortality was extracted from the record system regardless of blinding. Follow-up information was considered not to qualify for a star if information about it could not be located in the article.

**Table 2:** Quality assessment of RCTs through Cochrane Collaboration tool

Bias type	Selection		Performance	Detection	Attrition	Reporting
Study, year	Random sequence generation	Allocation concealment	Blinding of participants and personnel	Blinding of outcome assessment	Incomplete outcome data assessments	Selective reporting
Sharma et al. 2022 [37]	Low	Unclear	High	Low	Low	Low
Bauer et al. 2021 [38]	Unclear	Low	High	Low	Low	Low
Cohen et al. 2020 [39]	Low	Low	High	Low	Low	Low
Lopes et al. 2021 [40]	High	Unclear	Low	Low	Low	Low

Low, high and unclear denote low risk, high risk and unclear risk of bias judged by the related information

**Table 3:** Quality assessment of the included cohort studies by Newcastle–Ottawa scale

Study, year	Selection				Comparability	Outcome			Overall stars
	Representativeness of the exposed cohort	Representative of the non-exposed cohort	Ascertainment of exposure	Outcome was not present at start of study		Assessment of the outcome	Length of follow-up	Adequacy of follow-up	
Cannata et al. 2020 [30]	★	★	★	★	★	★	★	★	8
Lam et al. 2020 [31]	★	★	★	★	★	★	–	–	6
Chaudhri et al. 2020 [32]	★	★	★	★	★	★	–	–	6
Soleimani et al. 2020 [33]	★	★	★	★	★	★	★	★	8
Lahens et al. 2021 [34]	★	★	★	★	★	★	★	★	8
Aparisi et al. 2022 [35]	★	★	★	★	★	–	–	–	5
de Abajo et al. 2021 [36]	★	★	★	★	★	★	★	★	8

★: the quality of this specific item met the criterion; –: the item was not qualified to be awarded a star

### Effect of ACEI/ARB continuation on hospitalization and 30-day mortality

**Fig. 2:**
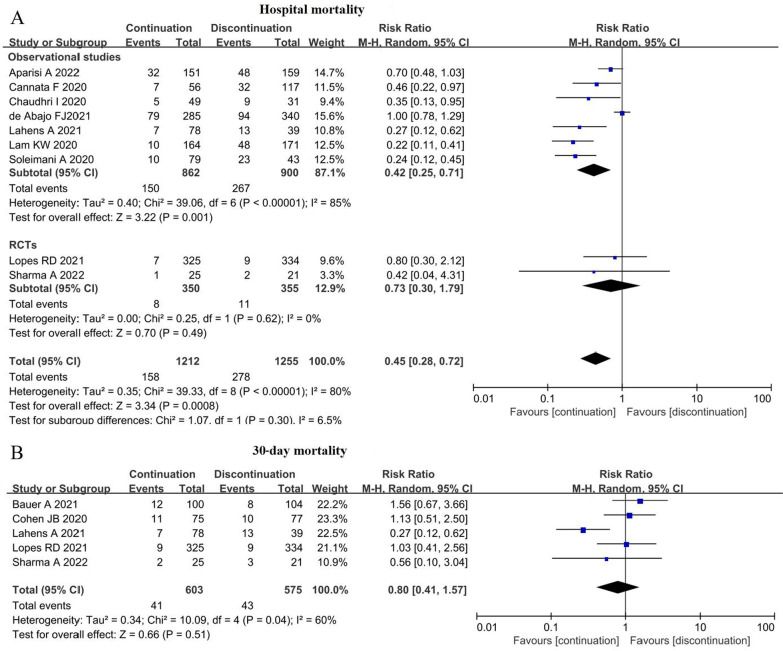
Effect of ACEI/ARB continuation on the hospital and 30-day mortality. **A** Effect of ACEI/ARB continuation on the hospital mortality; **B** effect of ACEI/ARB continuation on the 30-day mortality. *CI* confidence interval, *M–H* Mantel–Haenszel, *RCT* random controlled trial

Nine studies covering 2467 patients, reported hospital mortality, and the pooled hospital mortality was 13.04% (158/1212) and 22.15% (278/1255) in the continuation group and discontinuation group respectively, and the difference was significant in random effect model (RR = 0.45; 95% CI 0.28–0.72; z = 3.34, P = 0.0008) with high heterogeneity among studies (I^2^ = 80%; P < 0.00001) (Fig. 2A),sensitivity analysis demonstrated that the difference was still significant in fixed effect model (RR = 0.60; 95% CI 0.51–0.71; z = 5.83, P < 0.00001). Five studies, recruiting 1178 patients, reported on 30-day mortality, and according to the pooled results, no significant difference was observed between the continuation and discontinuation of ACEIs/ARBs (6.8% versus 7.5%; RR, 0.83; 95% CI 0.56–1.22; z = 0.96; I^2^ = 51%; P = 0.34) (Fig. 2B).

### Effect of ACEI/ARB continuation on ICU admission and IMV use

**Fig. 3:**
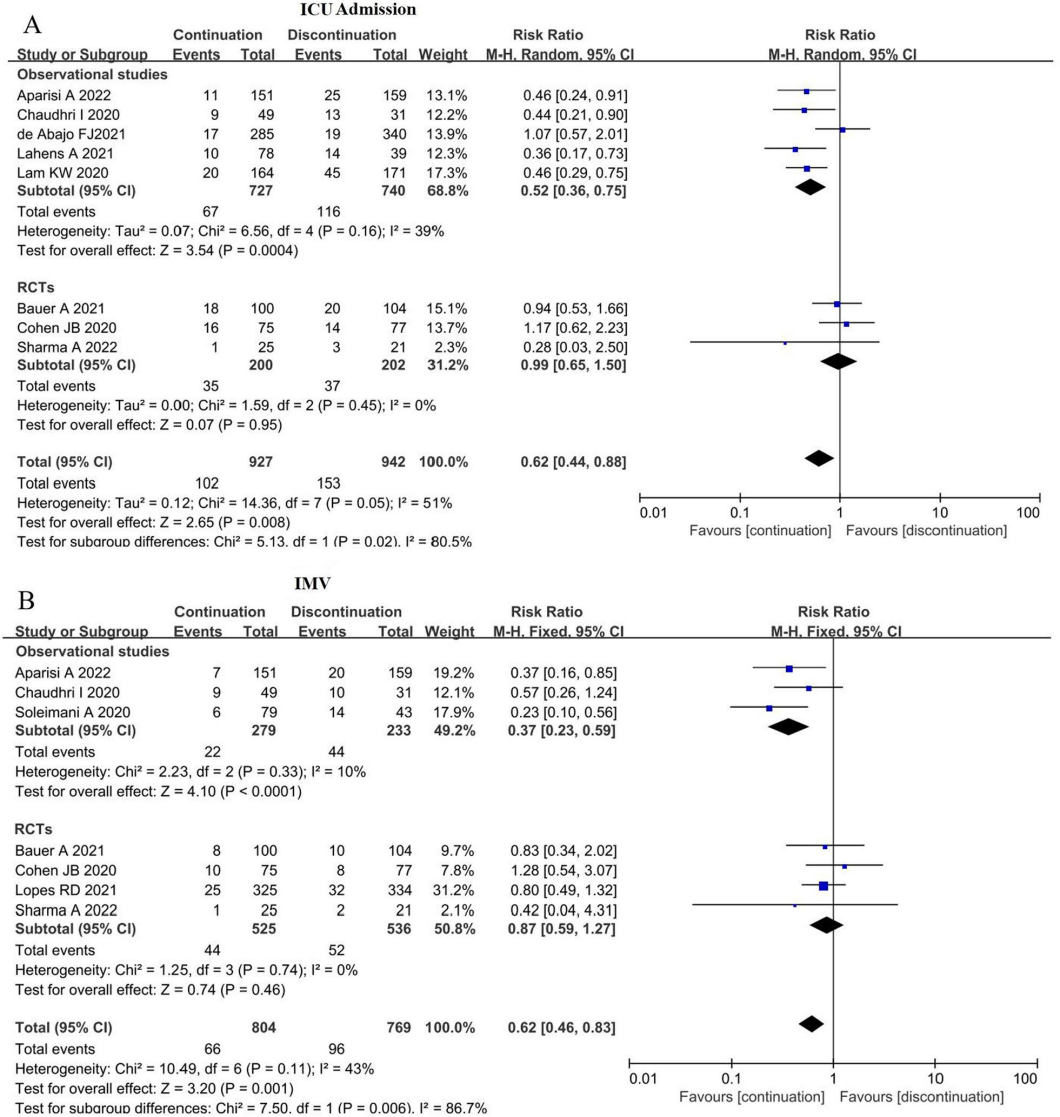
Effect of ACEI/ARB continuation on ICU admission and invasive mechanical ventilation. **A** Effect of ACEI/ARB continuation on ICU admission; **B** effect of ACEI/ARB continuation on the rate of IMV. *ICU* intensive care unit, *IMV* invasive mechanical ventilation, *CI* confidence interval, *M–H* Mantel–Haenszel

As shown in Fig. 3A, the pooled results indicate that the ICU admission rate was lower in the ACEI/ARB continuation group (10.5%, 102/927) than in the ACEI/ARB discontinuation group (16.2%, 153/942), and the difference between the two groups was statistically significant (RR = 0.63; 95% CI 0.5–0.79; z = 3.94; P < 0.0001) with moderate heterogeneity (I^2^ = 51%; P = 0.05). Moreover, the continuation of ACEIs/ARBs also was associated with the lower rate of IMV (8.2% versus 12.5%; RR = 0.62; 95% CI 0.46–0.83; P = 0.001) with low heterogeneity (I^2^ = 43%; P = 0.11) (Fig. 3B).

### Effect of ACEI/ARB continuation on key organs

**Fig. 4:**
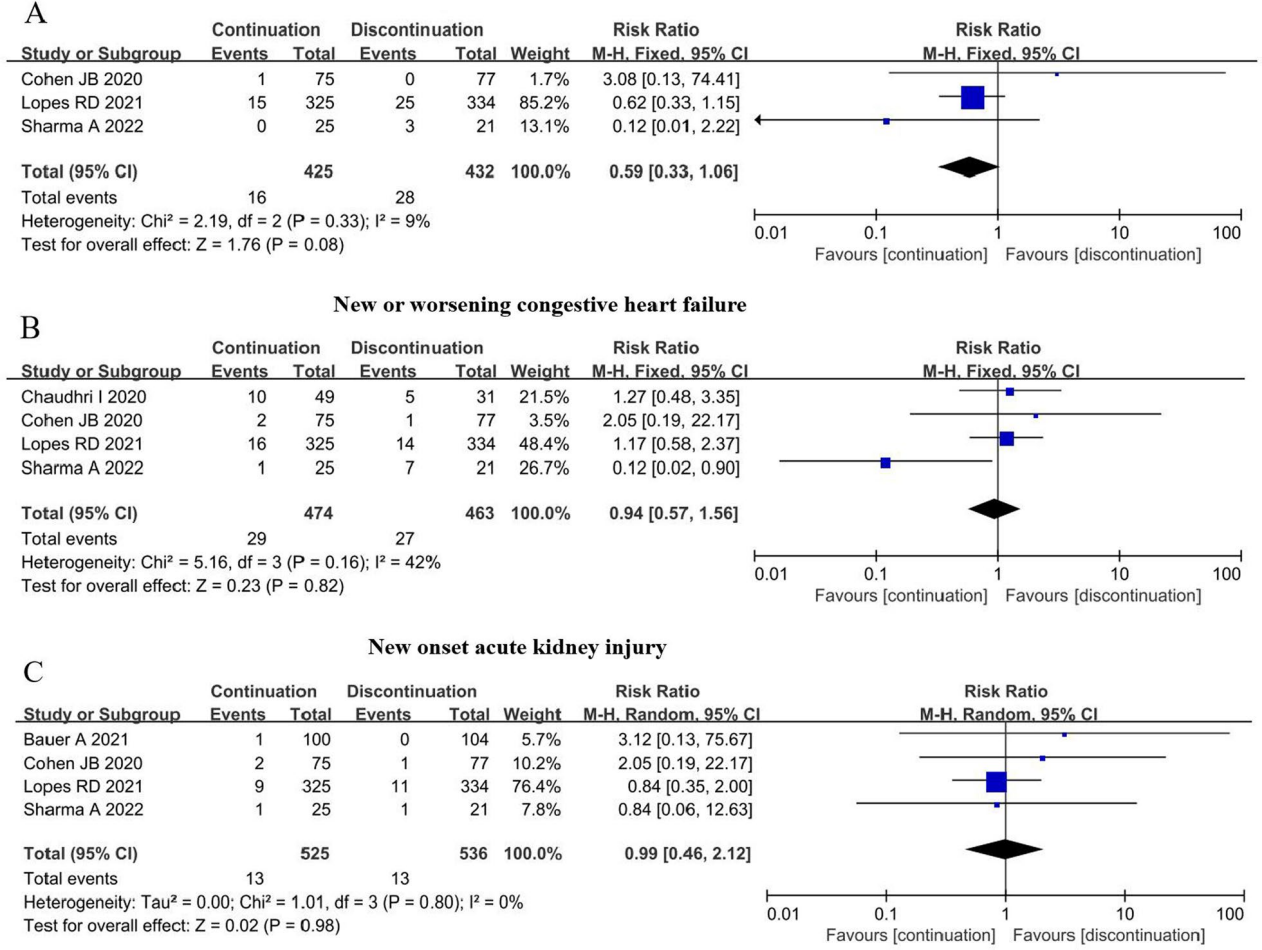
Effect of ACEI/ARB continuation on heart and kidney

A few included studies reported the outcomes of key organs. The pooled results indicated that ACEI/ARB continuation did not have significant effects on acute myocardial infarction, new or worsening congestive heart failure, and new onset acute kidney injury (P = 0.08, P = 0.82, and P = 0.98, respectively, Fig. 4).

### Subgroup and sensitivity analysis

Subgroup analysis based on the research design (RCT or observational study) revealed a significant effect of ACEI/ARB continuation, predominantly in observational studies (RR 0.42; 95% CI 0.25–0.71; P = 0.001), and the pooled in-hospital mortality was higher in observational studies than in RCTs in both the intervention (17.4% versus 2.3%) and control (29.7% versus 3.1%) groups. The benefit in reducing ICU admission and IMV use mainly derives from observational studies (P < 0.05). Subgroup analysis based on the number of research centers (single or multiple centers) revealed that the benefit of ACEI/ARB continuation was predominantly observed in single-center studies with low heterogeneity (RR 0.20; 95% CI 0.28–0.39; P < 0.00001; I^2^ = 0%) (Additional file 1: Fig. S1). According to the description about blood pressure (Additional file 1: Table S1), we allocated the studies into three categories: studies in which patients had comparable blood pressure between the two groups, studies in which some of the patients in the discontinuing group had hypotension, and studies in which patients had unstated blood pressure (Additional file 1: Table S2). Benefit in hospital mortality, rate of ICU admission, and IMV use was found in the studies in which some of the patients in the discontinuing group had hypotension (Table 4). Information about AKI and chronic kidney diseases was reported in Additional file 1: Table S3, unfortunately, it was not feasible to perform a successful analysis because of the incomplete data.

**Table 4:** Subgroup analysis by the baseline blood pressure

	No. of studies	Con event/total	Discon event/total	I^2^ (%)	*P* value for heterogeneity	RR 95% CI	Overall effect Z/*P* value
Hospital mortality subgroup analysis by blood pressure							
With comparable blood pressure	3	13/399	220/386	0	0.50	0.52 [0.27, 1.02]	1.9/0.06
Part of patients with hypotension in the discontinuing group	3	27/321	84/253	0	0.92	0.24 [0.16, 0.35]	7.07/< 0.00001
With unstated blood pressure	3	118/492	174/616	0	0.18	0.76 [0.52, 1.13]	1.35/0.18
30-day mortality subgroup analysis by blood pressure							
With comparable blood pressure	4	34/525	30/536	0	0.74	1.15 [0.72, 1.84]	0.59/0.56
Part of patients with hypotension in the discontinuing group	1	7/78	13/39	NA	NA	0.27 [0.12, 0.62]	3.08/< 0.002
Rate of ICU admission subgroup analysis by blood pressure							
With comparable blood pressure	4	44/249	50/233	42	0.16	0.76 [0.45, 1.28]	1.02/0.31
Part of patients with hypotension in the discontinuing group	2	30/242	59/210	0	0.55	0.43 [0.29, 0.64]	4.18/< 0.0001
With unstated blood pressure	2	28/436	44/499	68	0.08	0.71 [0.31, 1.51]	0.83/0.41
Rate of IMV subgroup analysis by blood pressure							
With comparable blood pressure	5	53/574	62/567	0	0.71	0.81 [0.57, 1.14]	1.21/0.23
Part of patients with hypotension in the discontinuing group	1	6/79	14/43	NA	NA	0.23 [0.10, 0.56]	3.24/0.001
With unstated blood pressure	1	7/151	20/159	NA	NA	0.62 [0.46, 0.83]	3.20/0.001

*Con* continuation, *Dis* discontinuation, *NA* not applicable, *ICU* intensive care unit, *IMV* invasive mechanical ventilation, *RR* risk ratio, *CI* confidence interval

Sensitivity analysis demonstrated that removing de Abajo et al. [36] (higher mortality rates but fewer patients admitted to ICU in both the intervention and control group) sharply decreased heterogeneity, and the reduction in hospital mortality rate remained statistically significant (RR 0.39; 95% CI 0.25–0.59; P < 0.0001; I^2^ = 58%) (Additional file 1: Fig. S2). For 30-day mortality, the heterogeneity (I^2^) decreased from 60% (P = 0.04) to 0 (P = 0.74) without distorting the pooled result (z = 0.59, P = 0.56) when one study [34] was removed. The heterogeneity of IMV use was attributed to the study performed by Abbas Soleimani [33], the origin of heterogeneity might be that the rate of IMV was vastly different in the aforementioned two studies. Adjusted RRs were reported in Additional file 1: Table S4, the differences of 30-day mortality and new or worsening congestive heart failure increased to be statistically significant (both Ps < 0.05).

### Meta-regression analysis

Meta-regression analysis was performed to explore the relationship between hospital mortality and the characteristics of the research design and patient baseline characteristics. The RR was found to be significantly affected by the sample size (t = 3.04, P = 0.019) (Additional file 1: Fig. S3A) but not by the publishing year (t = 1.29, P = 0.237) (Additional file 1: Fig. S3B), trial type (t = − 0.30, P = 0.771) (Additional file 1: Fig. S3C), number of research centers (t = 1.14, P = 0.292) (Additional file 1: Fig. S3D), and number of nations (t = − 1.91, P = 0.098) (Additional file 1: Fig. S3E). Multivariate meta-regression indicated these research design characteristics and explained the R^2^ = 78.78% heterogeneity result among studies on hospital mortality. Univariate meta-regression analysis revealed that the effect of patient baseline characteristics (age, male sex, rate of underlying chronic kidney disease or diabetes) on the association between ACEI/ARB continuation and hospital mortality was not significant (P > 0.05) (Additional file 1: Fig. S4); however, multivariate meta-regression revealed a confident model between hospital mortality and male sex (P = 0.023), and underlying diabetes (P = 0.023).

### Publication bias

Based on visual inspection, the funnel plot was slightly asymmetric, thus indicating potential publication bias (Additional file 1: Fig. S5), which was significant when evaluated using Egger’s test (P = 0.042) and not significant when assessed using Begg’s test (P = 0.754).

## Discussion

To the best of our knowledge, this is the first meta-analysis study to determine whether ACEIs/ARBs should be continued in patients with previous ACEI/ARB use in both the intervention and control groups before COVID-19 infection. We found that the continuation of ACEIs/ARBs could be maintained as it was associated with lower hospital deaths, ICU admission, and IMV in patients with COVID-19 without significant side effects on other clinical outcomes, although the benefits of continuing previous ACEI/ARB use were mainly shown in observational studies.

The RAAS has become a cause of concern ever since ACE2 was considered the binding receptor of SARS-CoV during the 2003 SARS pandemic [41]. In this COVID-19 pandemic, ACE2 has been implicated again as the key passage of SARS-CoV-2 entry into the host cells but with a 10- to 20-fold higher affinity than SARS-CoV [42]. Additionally, ACE2 has not only been limited to being a receptor; it has also been found to play a crucial role in lung injury caused by both SARS-CoV [43] and SARS-CoV-2 [7]. The RAAS is involved in the process of lung injury through the regulation of the ACE2-Ang-(1–7)-Mas receptor-G protein pathway and/or ACE-Ang II-AT1R pathway [44]. The two homologs interact with each other; ACE2 hydrolyzes Ang II to Ang-(1–7) and whittles the ACE-Ang II-AT1R pathway [45], whereas ACE cleaves Ang I to produce Ang II and counteracts the function of the ACE2-Ang II-Ang-(1–7) access. The imbalance of the two pathways dominates the effect of the RAAS during inflammatory lung injury [46, 47], thus rendering it possible to adopt ACEIs/ARBs or recombinant ACE2 protein as a therapeutic option [48]. Results of animal studies have found that ACEIs potentially reduce Ang-II and increase Ang-(1–7) levels in the plasma and ARBs potentially increase Ang-II, Ang-(1–7) levels, as well as the activity of ACE2. Therefore, ACEIs/ARBs potentially attenuate lung injury by hindering the classical RAAS axis (ACE-Ang II-AT1R) and aggrandizing the new ACE2-Ang II-Ang-(1–7)/Mas receptor pathway [10, 49].

At the clinical level, trials that explored the effect of ACEIs/ARBs on COVID-19-induced lung injury were predominantly performed on patients with hypertension before SARS-CoV-2 infection. Two cohort studies with large sample sizes indicated that taking ACEIs/ARBs before COVID-19 infection could reduce all-cause mortality compared with non-use of ACEIs/ARBs [9, 50]. However, a few meta-analyses found that taking ACEIs/ARBs did not affect mortality significantly [16, 51–54], but all the included trials compared the effect of ACEIs/ARBs with that of placebos or other anti-hypertension drugs. For these trials, it remains impeachable to draw a conclusion regarding the continuation or discontinuation of previous ACEI/ARB treatment considering the differing ACE2 levels between the two comparison groups. Patients in the control group took placebos or other antihypertensives, and ACE2 expression was not affected by ACEIs/ARBs, whereas ACE2 in intervention group patients was upregulated by ACEIs/ARBs.

The present study further clarified whether ACEIs/ARBs should be continued in patients who possessed the same level of ACE2 in both the intervention and control groups because they all took ACEIs/ARBs before COVID-19 infection. This novelty distinguishes the present study from aforementioned studies. The pooled result of the present study indicated that continued exposure to ACEIs/ARBs was associated with a 41.1% decrease in all-cause hospital mortality, thus corroborating the protective survival effect of continued ACEI/ARB use. This protective effect may be attributed to the multiple inhibition function of ACEIs/ARBs on inflammation induced by the RAAS axis. ACEIs inhibit the conversion of Ang I to Ang II, and ARBs block the attachment of Ang II to AT1R, thus interrupting the ACE-Ang I-Ang II-AT1R inflammatory pathway, with ACEIs/ARBs simultaneously increasing the substrates (Ang I and Ang II) of ACE2. Additionally, ACEIs/ARBs increase ACE2 expression and enhance its activity [55], thus playing a protective role after infection [56]. The increased substrates (Ang I and Ang II) and the enhanced converzyme (ACE2) produce more Ang-(1–7), which strengthens the Ang-(1–7)-Mas receptor anti-inflammatory pathway. Hence, we deemed ACEI/ARB continuation to relieve lung injury and the cytokine storm through correcting the imbalance between the COVID-19-induced inflammatory and anti-inflammatory pathways. This standpoint was verified by the lower rate of ICU admission and lower risk of IMV in the ACEI/ARB continuation group. In patients who took ACEIs/ARBs prior to COVID-19 infection, continued ACEI/ARB use mitigated the severity of COVID-19 and reduced the risk of death. Nonetheless, heterogeneity among studies was high, and further sensitivity analysis found that it predominantly arose from the study by de Abajo et al. [36]. In that study, the definition of (dis)continuation of ACEIs/ARBs was more rigorous, and the patient inclusion criteria were more stringent; additionally, hospital mortality was considerably high in both the ACEI/ARB discontinuation and continuation groups.

Subgroup analysis revealed that the effect of continuing ACE/ARB use in reducing hospital mortality, ICU admission, and IMV was predominantly demonstrated in observational studies; all RCTs found no difference between the continuation and discontinuation use of ACE/ARB. According to the strength of evidence, RCT provides more robust evidence than observational studies. There might be some confounders in the nonrandom trials. The reasons of discontinuing were excavated. Three studies reported that the reasons of ACEI/ARB discontinuation were dependent on clinical need such as hypotension and acute kidney injury [31, 33, 34]. Subgroup analysis was performed on blood pressure and found that the benefits of discontinuation were mainly demonstrated in the studies in which patients in the group who were discontinuing ACEI/ARB had hypotension, which is consistent with clinical medicine. However, it is well known that those who have severe symptoms have a higher risk of bad outcomes due to their serious condition besides the effect of the discontinuation of drugs, which hints that hypotension might be a vital confounding bias that could give misleading results, although baseline blood pressure and kidney function were comparable in all the included RCTs and one observational studies (Additional file 1: Table S2). Nevertheless, the weakness of this evidence cannot be used to give a recommendation to discontinue previous ACEI/ARB treatment as no evidence was found that indicated a disadvantage in continuing ACEI/ARB use [57]. In addition, blind withdrawal of drugs may result in unstable disease control [58, 59]. As a result, ACEI/ARB treatment could be maintained based on the current results of the present study. However, more RCTs should be performed to obtain more robust evidence.

Meta-regression indicated that male sex and diabetes were impact factors, a finding that is inconsistent with that of studies in which the pooled results compared ACIEs/ARBs with placebos or other antihypertensive agents [51]. The negative impact of male sex might have been related to the higher expression of ACE2 [60], which allowed more SARS-CoV-2 to enter the patients’ body and cause more severe disease and higher mortality than that in the female sex [61, 62]. Similarly, ACE2 expression was upregulated approximately 30% in both type 1 [63] and type 2 [64] diabetes. The mechanism underlying the negative effect of diabetes might not have been limited to the higher level of ACE2; a more complicated pathophysiology might have been involved in the disease course of patients COVID-19 and comorbid diabetes [65].

### Limitations

Our conclusions were predominantly derived from observational studies, although they were confirmed to be of relatively high quality. More RCTs were required to strengthen the evidence. ACEIs and ARBs elicit different feedbacks in the RAAS, especially the levels of Ang II, Ang-(1–7) ACE, and ACE2 [4], thus potentially making them exert varying effects on inflammation and even the clinical outcomes. However, this study had no means of clarifying this because subgroup analysis based on antihypertensive agents, ACIs, or ARBs could not be performed. The findings might be affected to a certain extent by a confounder (patients had more severe symptoms in the ACEI/ARB discontinuation group). There was no way to elucidate the influence of this confounding factor by multivariate regression analysis with the effect size because limited data could be extracted from the included trials in this meta-analysis study, however, sensitivity analysis by removing a dataset, changing the effect model or introducing adjusted RR as effect size indicated robust and stable findings in most of the outcomes of interest. Additionally, publication bias might have subsisted owing to the divergence between the Egger’s and Begg’s tests.

## Conclusions

In patients with COVID-19, previous ACEI/ARB treatment could be continued as the continuation of ACEI/ARB use was associated with reduced hospital mortality, ICU admission, and IMV, without remarkable disadvantage. However, the benefits were mainly shown in observational studies, and simultaneously, the potential confounders should be kept in mind. The effects of the continuation of ACEI/ARB treatment might have also been affected by sample size, male sex, and underlying diabetes mellitus. More multicenter RCTs are warranted to enhance the robustness of evidence.

## Supplementary Information

**Additional file 1: Figure S1.** Subgroup analysis on hospital mortality according to the number of research centers. **Figure S2.** Sensitivity analysis of heterogeneity among studies on hospital mortality. **Figure S3.** Meta-regression analysis to find the impact factor of ACEI/ARB continuation on hospital mortality. **Figure S4.** Univariate meta-regression analysis to find the impact factor of ACEI/ARB continuation on in-hospital mortality. **Figure S5.** Funnel plots to estimate the publication bias regarding the effect of ACEI/ARB continuation on hospital mortality. **Table S1.** Reasons of discontinuing ACEi/ARB. **Table S2.** Blood pressure of the included patients. **Table S3.** Baseline acute kidney injury and chronic kidney disease. **Table S4.** The pooled adjusted RR of the outcomes of interest.

## Data Availability

The dataset supporting the conclusions of this article is available in the personal homepage of the corresponding author, Q.L., at ResearchGate (https://www.researchgate.net/profile/Qi-Liu-169).

## Abbreviations

RAAS: Renin–angiotensin–aldosterone system
ACE2: Angiotensin-converting enzyme 2
ACE: Angiotensin-converting enzyme
Ang I: Angiotensin I
Ang II: Angiotensin II
AT1R: Type 1 angiotensin receptor
SARS-CoV-2: Severe acute respiratory syndrome coronavirus 2
NOS: Newcastle–Ottawa Scale
COVID-19: Corona virus disease 2019
ACEIs: Angiotensin-converting enzyme inhibitors
ARBs: Angiotensin receptor blockers
CKD: Chronic kidney disease
ICU: Intensive care unit
RR: Risk ratio
CI: Confidence interval
RCT: Randomized controlled trial
