# Supplementary material for: Effect of continuing the use of renin–angiotensin system inhibitors on mortality in patients hospitalized for coronavirus disease 2019: a systematic review, meta-analysis, and meta-regression analysis
Source: BMC Infect Dis. 2023 Jan 24;23:53. doi: 10.1186/s12879-023-07994-7 (PMC9872739; doi:10.1186/s12879-023-07994-7)
Supplement: Supplementary file 1 — Additional file 1: Figure S1. Subgroup analysis on hospital mortality according to the number of research centers. Figure S2. Sensitivity analysis of heterogeneity among studies on hospital mortality. Figure S3. Meta-regression analysis to find the impact factor of ACEI/ARB continuation on hospital mortality. Figure S4. Univariate meta-regression analysis to find the impact factor of ACEI/ARB continuation on in-hospital mortality. Figure S5. Funnel plots to estimate the publication bias regarding the effect of ACEI/ARB continuation on hospital mortality. Table S1. Reasons of discontinuing ACEi/ARB. Table S2. Blood pressure of the included patients. Table S3. Baseline acute kidney injury and chronic kidney disease. Table S4. The pooled adjusted RR of the outcomes of interest. [file 12879_2023_7994_MOESM1_ESM.doc]

**Effect of continuing the use of renin–angiotensin system inhibitors on mortality in patients hospitalized for coronavirus disease 2019: a systematic review,**

**meta-analysis, and meta-regression analysis**

**Figure S1 Subgroup analysis on hospital mortality according to the number of research centers**


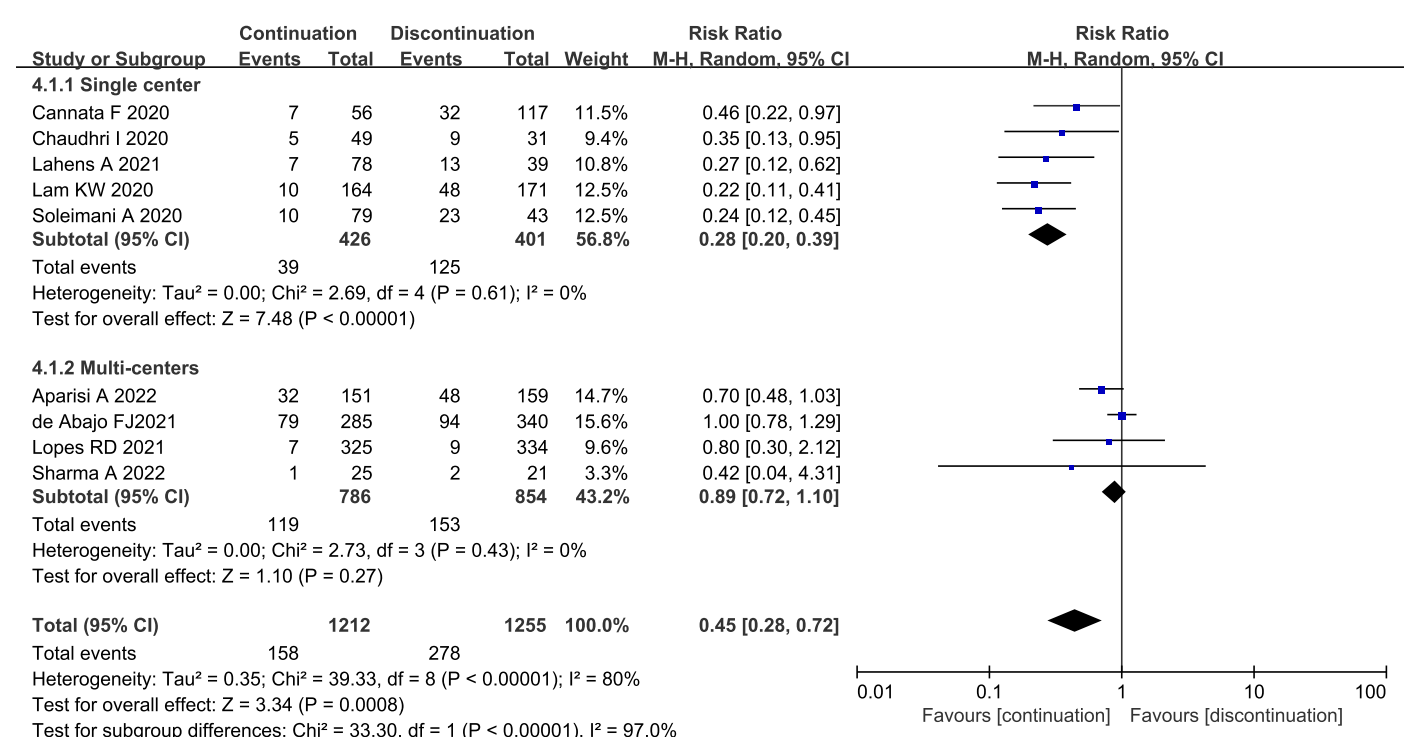


**Figure S2 Sensitivity analysis of heterogeneity among studies on hospital mortality**


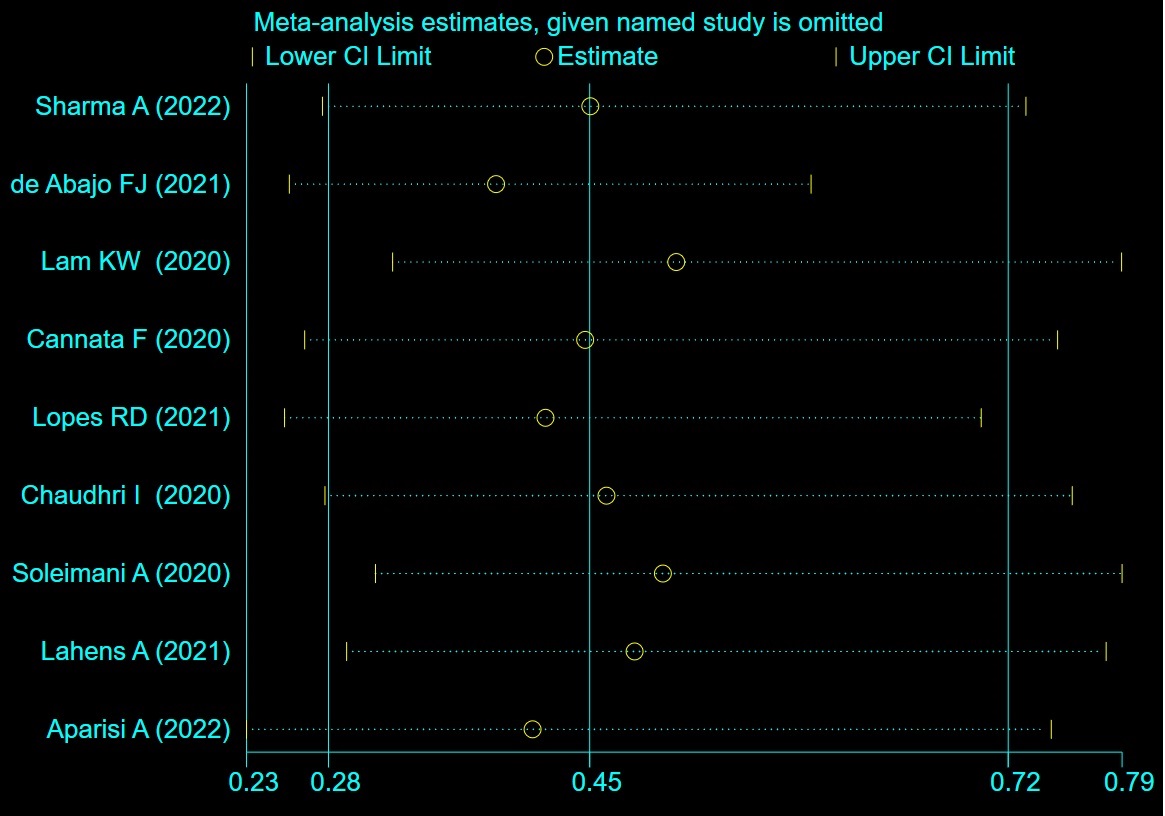


------------------------------------------------------------------------------

Study omitted | Estimate [95% Conf. Interval]

-------------------+----------------------------------------------------------

Sharma A (2022) | .44802582 .27546379 .72868794

de Abajo FJ (2021)| .38729891 .25412282 .59026754

Lam KW (2020) | .50340563 .32065114 .79032063

Cannata F (2020) | .44470975 .26401767 .74906635

Lopes RD (2021) | .41916832 .25103551 .69990927

Chaudhri I (2020)| .45843139 .27706543 .75851882

Soleimani A (2020)| .49479505 .30965129 .79063821

Lahens A (2021) | .47656253 .29102781 .7803784

Aparisi A (2022) | .41082016 .22653446 .7450223

-------------------+----------------------------------------------------------

Combined | .44761777 .27933274 .71728674

------------------------------------------------------------------------------

The sensitivity analysis showed that removing de Abajo FJ (higher mortality but less patients admitted to ICU in both intervention and control group) would decrease the hetreogeneity sharply and the in-hospital mortality benefit was still statistically significant (RR 0.39; 95% CI, 0.25-0.59; p < 0.0001; I^2^=58%, p = 0.02)

**Figure S3**  **Meta-regression analysis to find the impact factor of ACEI/ARB continuation on hospital mortality**


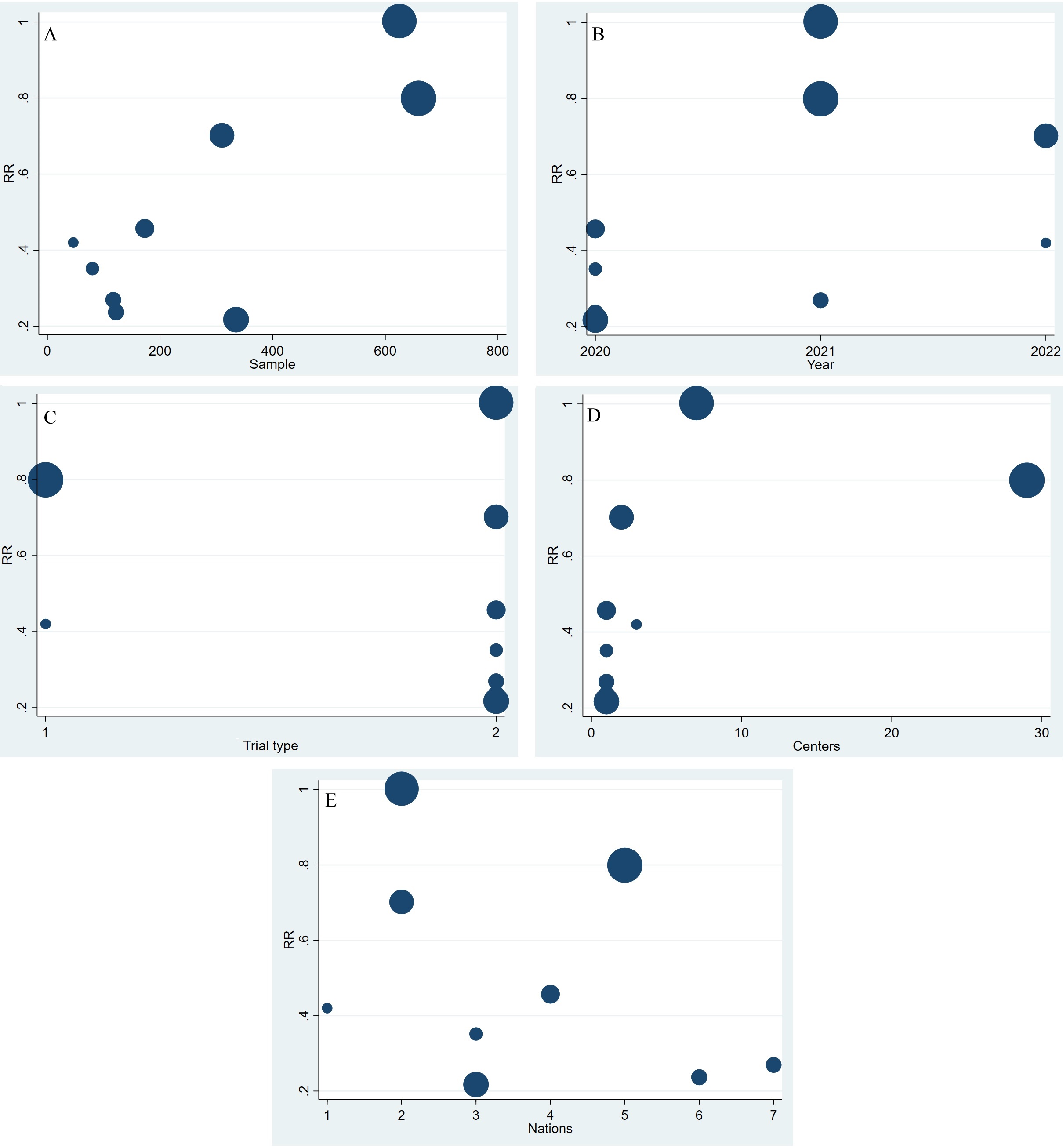


RR, risk ratio, was significantly affected by sample size (Figure S3A,t = 3.04, P = 0.019),but was non-significantly affected by the other covariates including publishing year (Figure S3B,t = 1.29, P = 0.237) , trial type (Figure S3C,t = -0.30, P = 0.771) , centers (Figure S3D,t = 1.14, P = 0.292), and the number of nations (Figure S3E,t = -1.91, P = 0.098) .

**Figure S4 Univariate meta-regression analysis to find the impact factor of ACEI/ARB continuation on in-hospital mortality**


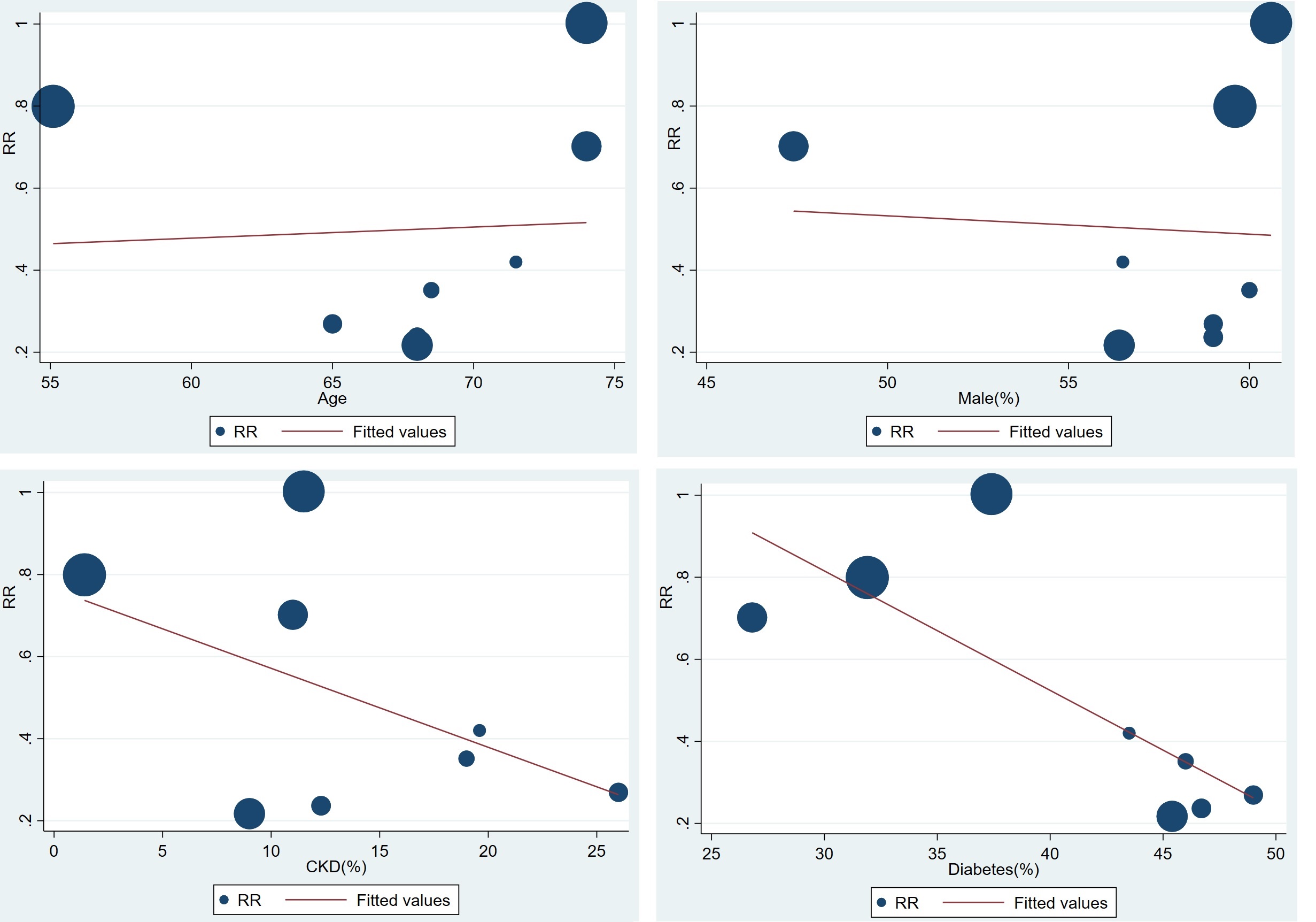


RR, risk ratio; ACEIs, angiotensin-converting enzyme inhibitors; ARBs, angiotensin receptor blockers; CKD,chronic kidney disease.

**Figure S5 Funnel plots to estimate the publication bias regarding the effect of ACEI/ARB continuation on hospital mortality**


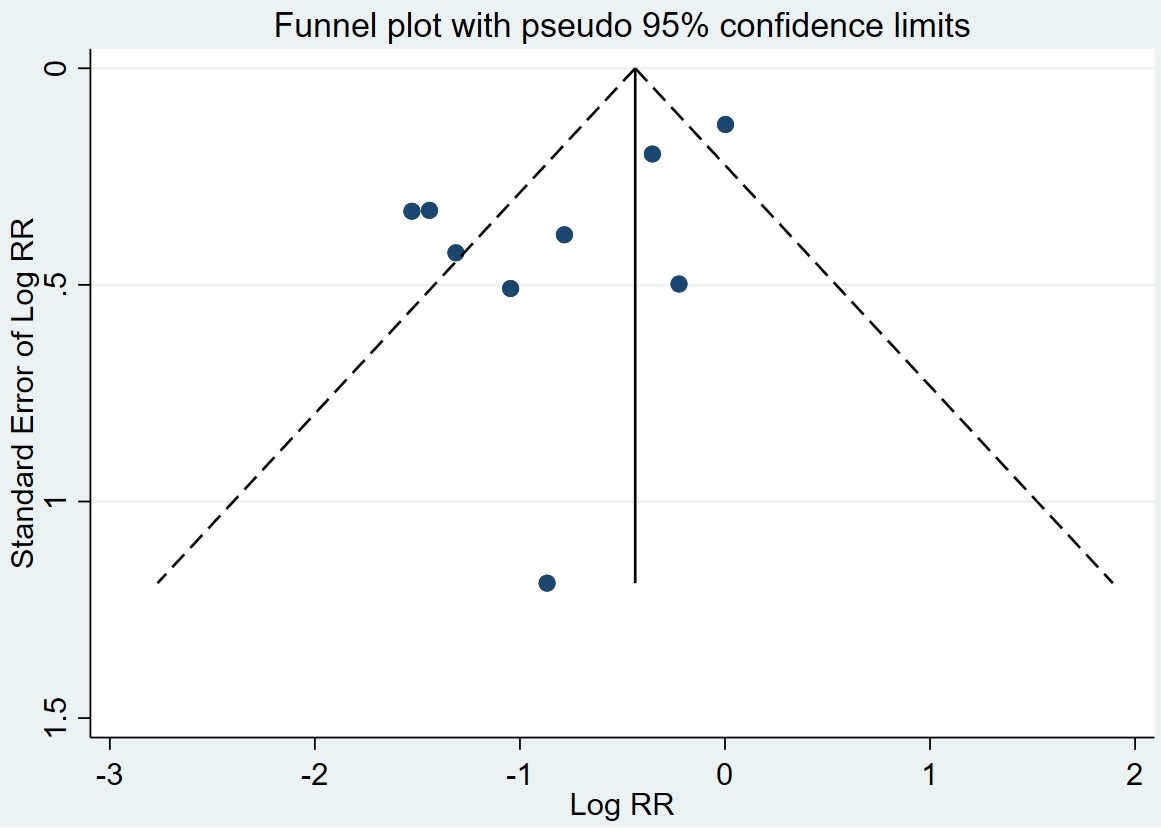


RR, Risk Ratio

**Table S1 Reasons of discontinuing ACEi/ARB**

| **Study Year** | **Reasons or explanations** |
| --- | --- |
| Cannata F 2020 [30] | Unaware of the reasons leading to ACEi/ARB discontinuation. |
| Lam KW 2020 [31] | the development of hypotension or AKI |
| Chaudhri I 2020 [32] | Not reported the reasons leading to ACEi/ARB discontinuation.  Blood pressure in the two groups were close to each other with 100% of hypertension at the time of admission |
| Soleimani A 2020 [33] | the inclusion in the trial in 23 (53.5%), both AKI and shock in 11 (25.6%), AKI in 6 (14.0%), and shock in 3 (7.0%) patients |
| Lahens A 2021 [34] | ICU admission or decision of palliative care in 16 patients, acute  kidney injury or hypotension in 20 patients, and was a seemingly arbitrary medical decision in three patients |
| Aparisi A 2022 [35] | No description |
| de Abajo FJ 2021 [36] | No description,but there was no significant difference in the baseline blood pressure |
| Sharma A 2022 [37] | RCT, Withdraw ACEi/ARB according to the pre-specified protocol not clinical need. Participants were excluded if they had a clinical indication  to stop ACEi or ARB treatment during hospitalization including hypotension or shock or immediate requirement for ventilation |
| Bauer A 2021 [38] | RCT, discontinuing ACEi/ARB according to the pre-specified protocol not clinical need. |
| Cohen JB 2021 [39] | RCT,  Discontinuing ACEi/ARB according to the pre-specified protocol not clinical need.  Participants were excluded if they had clinical contraindications to continuing or discontinuing ACEI or ARB therapy |
| Lopes RD 2021 [40] | RCT,  Discontinuing ACEi/ARB according to the pre-specified protocol not clinical need.  Patients with a clinical indication to stop ACEI or ARB treatment (eg, those with hypotension, acute kidney injury, or shock) also were excluded. |

ACEI,angiotensin converting enzyme inhibitor ; ARB,angiotensin receptor antagonist ;AKI，acute kidney injury; CKD，Chronic kidney disease; RCT,Randomized controlled trial；ICU，intensive care uint;

**Table S2 Blood pressure of the included patients**

| **Study Year** | **Systolic blood pressure**  **（mmHg）** | | **P Value** | **Diastolic blood pressure（mmHg）** | | **P Value** | **Mean arterial pressure** | | **P Value** | **Categories of patients according to the blood pressure** |
| --- | --- | --- | --- | --- | --- | --- | --- | --- | --- | --- |
|  | **Con** | **Dis** |  | **Con** | **Dis** |  | **Con** | **Dis** |  |  |
| Cannata F 2020 [30] | NA | NA | NA | NA | NA | NA | NA | NA | NA | With unstated blood pressure |
| Lam KW 2020 [31] | NA | NA | NA | NA | NA | NA | NA | NA | NA | Part of patients with hypotension in the discontinuing group |
| Chaudhri I 2020 [32] | 127.6±23.1 | 130.4±26.0 | 0.62 | 71.5±11.9 | 69.5±12.0 | 0.48 | 90.0±14.0 | 89.1±13.8 | 0.79 | With comparable blood pressure |
| Soleimani A 2020 [33] | NA | NA | NA | NA | NA | NA | NA | NA | NA | Part of patients with hypotension in the discontinuing group |
| Lahens A 2021 [34] | NA | NA | NA | NA | NA | NA | NA | NA | NA | Part of patients with hypotension in the discontinuing group |
| Aparisi A 2022 [35] | NA | NA | NA | NA | NA | NA | NA | NA | NA | With unstated blood pressure |
| de Abajo FJ 2021 [36] | NA | NA | NA | NA | NA | NA | NA | NA | NA | With unstated blood pressure |
| Sharma A 2022 [37] | 135.7 ±19.2 | 136.7 ±17.0 | NS | 71.6 ±9.6 | 71.0 ±11.8 | NS | NA | NA | NA | With comparable blood pressure  Participants were excluded if they had clinical contraindications to continuing or discontinuing ACEI or ARB therapy |
| Bauer A 2021 [38] | 130 (120–140) | 130 (120–141) | NS | 75 (70–80) | 77 (68–80) | NS | NA | NA | NA |
| Cohen JB 2021 [39] | 129±19 | 133 ±22 | NA | 75±13 | 77±12 | NA | NA | NA | NA |
| Lopes RD 2021 [40] | 135.0 (125.0-149.0) | 139.0 (125.0-149.0) | NA | NA | NA | NA | NA | NA | NA |

Con, continuation; Dis,discontinuation;NA，not available; NS, non-significant

**Table S3 Baseline acute kidney injury and chronic kidney disease**

| **Study Year** | **Item of kidney** | **Con n(%)** | **Dis n(%)** | **P Value** |
| --- | --- | --- | --- | --- |
| Cannata F 2020 [30] | NA | NA | NA | NA |
| Lam KW 2020 [31] | AKI | 5(16.12) | 35 (34.65) | NA |
| Chaudhri I 2020 [32] | AKI | 12 (25) | 18 (58) | 0.003 |
|  | CKD | 8 (16) | 7 (23) | 0.49 |
| Soleimani A 2020 [33] | AKI | 14 (17.7) | 17 (39.5) | ˂0.00625 |
| Lahens A 2021 [34] | NA | NA | NA | NA |
| Aparisi A 2022 [35] | NA | NA | NA | NA |
| de Abajo FJ 2021 [36] | Chronic renal failure | 34 (11.9) | 38 (11.2) | 0.77 |
| Sharma A 2022 [37] | CKD | 5 (20.0) | 4 (19.1) | NA |
| Bauer A 2021 [38] | CKD | 21 (21) | 16 (15) | NA |
| Cohen JB 2021 [39] | NA | NA | NA | NA |
| Lopes RD 2021 [40] | Kidney disease | 4 (1.2) | 5 (1.5) | NA |

Con, continuation; Dis,discontinuation;NA,not available; AKI，acute kidney injury; CKD，Chronic kidney disease;

**Table S4** **The pooled adjusted RR of the outcomes of interest**

|  | No. of studies | Weight  (%) | I^2^  (%) | *P* Value for  heterogeneity | Adjusted RR  95% CI | Overall effect  Z / *P* Value |
| --- | --- | --- | --- | --- | --- | --- |
| **Hospital mortality** | | | | | | |
| Observational study subgroup | 7 | 95.8 | 79 | <0.0001 | 0.66[0.54, 0.81] | 4.09/<0.0001 |
| RCT subgroup | 2 | 4.2 | 0 | 0.81 | 0.87[0.38, 2.01] | 0.33/0.74 |
| Overall effect | 9 | 100 | 72 | 0.0003 | 0.67[0.55, 0.81] | 4.19/<0.0001 |
| **30-day mortality** | 5 | 100 | 0 | 0.51 | 0.62 [0.49, 0.78] | 4.1/<0.0001＊ |
| **ICU admission** | | | | | | |
| Observational study subgroup | 5 | 90.5 | 0 | 0.70 | 0.70[0.61, 0.81] | 4.94/<0.00001 |
| RCT subgroup | 3 | 9.5 | 0 | 0.82 | 0.94 [0.61, 1.45] | 0.29/0.77 |
| Overall effect | 8 | 100 | 0 | 0.80 | 0.72[0.63, 0.82] | 4.79/<0.00001 |
| **IMV** | | | | | | |
| Observational study subgroup | 3 | 79.9 | 19 | 0.29 | 0.59 [0.49, 0.70] | 5.84/<0.00001 |
| RCT subgroup | 4 | 20.4 | 0 | 0.98 | 0.92 [0.65, 1.30] | 0.48/0.63 |
| Overall effect | 7 | 100 | 20 | 0.28 | 0.64 [0.55, 0.75] | 5.42/<0.00001 |
| **Effect of ACEI/ARB continuation on heart and kidney** | | | | | | |
| Acute myocardial infarction | 3 | 100 | 0 | 0.49 | 0.75 [0.51, 1.09] | 1.5/0.13 |
| New or worsening congestive heart failure | 4 | 100 | 40 | 0.17 | 0.51 [0.35, 0.75] | 3.44/0.0006＊ |
| New onset acute kidney injury | 4 | 100 | 0 | 1 | 0.93 [0.41, 2.10] | 0.18/0.86 |
| RR,risk ratio; CI, confidence interval; RCT, randomized controlled trial; ICU, intensive care unit;IMV, invasive mechanical ventilation; ACEI,angiotensin converting enzyme inhibitor ; ARB,angiotensin receptor antagonist;＊  the differences increased to be statistically significant (both Ps <0.05) | | | | | | |
